# Developing a dementia care and support needs framework for Culturally and Linguistically Diverse populations: A whole‐of‐community co‐design approach

**DOI:** 10.1111/ajag.70031

**Published:** 2025-04-21

**Authors:** Nina Bala, Biljana Stanoevska, John Paul Troiani, Xinxia Wang, Nadine Veerhuis, Leissa Pitts, Victoria Traynor

**Affiliations:** ^1^ University of the Sunshine Coast Sippy Downs Queensland Australia; ^2^ Warrigal Oak Flats New South Wales Australia; ^3^ Formerly at the University of Wollongong Wollongong New South Wales Australia; ^4^ Illawarra Shoalhaven Local Health District Warrawong New South Wales Australia; ^5^ University of Wollongong Wollongong New South Wales Australia

**Keywords:** Australia, community networks, cultural diversity, dementia, health‐care disparities

## Abstract

**Objectives:**

Older individuals with dementia and their families from CALD backgrounds face a ‘triple jeopardy’ due to the combination of dementia, caregiving challenges and cultural stigma. Despite the growing need for culturally responsive dementia care, existing services do not adequately address the specific concerns of Culturally and Linguistically Diverse (CALD) communities. This study explored the experiences of two CALD communities to inform the development of a culturally tailored dementia care support framework.

**Methods:**

This study employed a whole‐of‐community co‐design approach, integrating community‐based participatory research and human‐centred design principles. A total of 36 participants, including nine individuals with dementia and 27 family caregivers from the Macedonian and Italian communities in the Illawarra Shoalhaven region of NSW, Australia, collaborated with ADHERe researchers and the Multicultural Health Service. Data were collected through co‐designed focus groups and interviews and analysed using thematic analysis. Participant feedback was regularly incorporated to ensure the framework reflected their lived experiences and needs.

**Results:**

Key barriers to dementia care included emotional and psychological strain contributing to caregiver burden, social isolation limiting peer support, language difficulties hindering communication with health‐care providers and cultural misunderstanding impacting service uptake. Participants emphasised the importance of face‐to‐face interactions, culturally relevant educational programs and practical guidance from health‐care professionals to improve dementia care and support.

**Conclusion:**

This study highlighted the need for a culturally tailored, community‐driven dementia care framework. Piloting a dementia education program with the Macedonian and Italian communities will provide valuable insights for expanding support to other CALD groups, promoting more inclusive and accessible dementia care.

**Policy impact**

Gaps in Illawarra's dementia care force CALD individuals to seek support elsewhere, highlighting the need for locally tailored interventions. Trust, continuity and culturally tailored in‐person education are essential for effective dementia care. Co‐designing dementia care initiatives with CALD communities ensures accessibility, overcomes barriers and empowers families to navigate services effectively.

## 1 INTRODUCTION

As Australia's population becomes increasingly multicultural, dementia care must adapt to meet the diverse linguistic and cultural needs of its communities. Currently, approximately 31% of Australian residents or 8.2 million people were born overseas.^1^ With this growing cultural diversity, the prevalence of dementia within Culturally and Linguistically Diverse (CALD) communities is also rising. In 2018, 28% of individuals living with dementia identified as being from CALD backgrounds, with nearly half (47%) relying solely on informal caregiving.^2^ These figures highlighted the urgent need for culturally responsive dementia care.

Older individuals with dementia and their families from CALD backgrounds face a ‘triple jeopardy’ due to the combined impact of dementia, caregiving challenges and cultural stigma.^3^ Language barriers hinder effective communication with health‐care providers, while financial constraints may limit access to translated materials and culturally tailored services.^4^ Additionally, cultural stigma discourages help‐seeking, as some communities perceive dementia as either a normal part of ageing or a source of shame.^3^ Many CALD communities prioritise home‐based care over residential aged care facilities.^5^ While this aligns with cultural values, it places significant stress on family caregivers, who must balance work, caregiving duties and navigating government support services, such as home care packages and dementia awareness programs.^6^

Australian dementia research does not adequately capture the diverse experiences of CALD communities.^7^ Although organisations like the National Health and Medical Research Council and the National Institute for Dementia Research acknowledge the need for inclusivity, CALD communities remain under‐represented.^8^ A study in Denmark found that dementia education programs led by multicultural workers trained in dementia care improved knowledge and encouraged help‐seeking, highlighting the potential of culturally tailored approaches.^9^ To address this gap in Australian research, this study adopted a co‐design approach to develop a dementia care and support framework, incorporating a dementia education program.^10^ Co‐design enables CALD individuals, caregivers and service providers to actively contribute to research, assisting in the creation of culturally appropriate and accessible dementia care strategies. This process ensures that the resulting framework is relevant, effective and responsive to the needs of CALD communities.^11^

### 1.1 Aim

This study employed a whole‐of‐community co‐design approach to develop a dementia care and support needs framework tailored to the Macedonian and Italian communities in the Illawarra Shoalhaven region of NSW, Australia.

### 1.2 Stakeholder contributions

This study was co‐initiated by researchers from the Aged Dementia Health Education and Research (ADHERe) organisation and the Multicultural Health Service (MHS) of the Illawarra Shoalhaven Local Health District. Multicultural Health Officers (MHOs) from MHS, community representatives and ADHERe researchers collaboratively co‐designed the study's aim, design, data collection methods and analysis approaches. This process was facilitated through a series of structured consultations, including community meetings, advisory group discussions and iterative feedback sessions.

## 2 METHODS

This research was guided by Community‐Based Participatory Research (CBPR) and Human‐Centred Design (HCD), which informed the whole‐of‐community co‐design approach. Community‐Based Participatory Research emphasises equitable collaboration between researchers and communities, ensuring cultural appropriateness, community ownership and alignment with the lived experiences and priorities of CALD individuals.^12^ Human‐Centred Design complements this by actively engaging stakeholders throughout the research process to develop practical, meaningful solutions.^12^ These frameworks were selected to ensure an inclusive research process that reflects community needs and generates actionable insights for dementia care in CALD populations. The study adhered to the University of Wollongong Consolidated Criteria for Reporting Qualitative Studies guidelines, and ethics approval was obtained from the University of Wollongong Human Research Ethics Committee (2022/ETH00046).

### 2.1 Sample and setting

This study included individuals living with dementia and their family caregivers, such as adult son or daughter and extended family members, from Macedonian and Italian backgrounds. These two CALD communities were identified as priority groups by Multicultural Health Service due to their significant presence in the region and specific health‐care needs.^13^ Participants ranged in age from 23 to 93 years, providing multi‐generational insights from younger caregivers and older individuals living with dementia. Since no individuals younger than 18 years were approached or included in this study, parental or guidance consent was not required.

Participants were recruited through convenience sampling via established community groups that had ongoing relationships with MHOs.^14^ Recruitment was facilitated by a female Macedonian MHO and a male Italian MHO, both fluent in their native languages and English. Additional recruitment occurred through word‐of‐mouth and social networks, allowing participants to refer others from their communities.

### 2.2 Data collection

Preliminary consultations with MHOs and community members shaped the study design and this ensured the research questions, data collection methods and analysis approaches were relevant and culturally appropriate. The preferences of MHOs were respected and ensured a culturally responsive and participant‐driven research process. The sessions, lasting 45 and 60 min, were facilitated by the MHOs using a semi‐structured interview guide (Table 1) that was co‐developed through an iterative process. Draft questions were reviewed and refined based on feedback from the community members.

**TABLE 1:** Interview guide for focus groups and interviews.

Question	
1.	What questions did you have when the diagnosis was first disclosed to you? and were they answered? (If the answer is no, if this can be done again, how would you like health‐care professionals to explain to you?)
2.	What are your experiences to tell someone who takes care of people with dementia (positive or negative) (daily routine)?
3.	What would you suggest an education program look like? (do not mention face to face or online at the beginning, see them as prompt questions)
4.	What are your thoughts regarding online forums for information or support?
5.	Is there anything that you would like to mention regarding developing a Dementia Care and Support Needs education program? What are they?
6.	Would you like to say anything further regarding our previous questions? (Repeat the previous questions, if necessary. Allow the participants in the focus group to think at least 2–3 mins for each question, if possible)

To support informed participation, information sheets and consent forms were translated to the preferred languages of the participants. Multicultural Health Officers facilitated the consent process by providing verbal explanations for those individuals who needed further translation. Consent was obtained through written signatures and verbal confirmation. For participants living with dementia, particularly those in later stages, additional support was provided to ensure comprehension and voluntary participation. This included assessing their understanding, engaging a family member when necessary and adopting a person‐centred approach to ensure the individual's autonomy were promoted.

All sessions were audio‐recorded and transcribed verbatim by the MHOs. Field notes were also used to record non‐verbal cues and contextual insights to enhance interpretation of the data. De‐identified data were securely stored with restricted access to maintain confidentiality. Regular debriefing sessions between the researchers and MHOs enabled reflexivity and minimise biases.^15^ Data collection concluded upon reaching thematic saturation.

### 2.3 Data analysis

Data analysis followed Braun and Clarke's six‐phase thematic analysis, using an inductive approach to ensure the findings accurately reflected participants' lived experiences.^16^ The transcripts were independently manually coded by an ADHERe researcher and two MHOs. The coded data were reviewed and refined through consensus discussions involving community members, who played an integral role in validating the emerging themes. These discussions helped ensure that the themes aligned with community priorities and accurately reflected the cultural contexts of the Macedonian and Italian communities.

Given the language barriers, verbal discussions were prioritised over written transcripts. Multicultural Health Officers facilitated these discussions, ensuring participants' perspectives were accurately captured and incorporated into the iterative review process. A member‐checking process was integrated, where emerging themes were shared with participants in follow‐up sessions to validate and refine the findings.^11^ Participants were invited to clarify any misunderstandings, suggest modifications and provide further insights, which were crucial for ensuring that the final themes were consistent with their views. Throughout the analysis, particular attention was given to highlighting both shared and distinct experiences between the Macedonian and Italian communities, providing a deeper understanding of the diversity within CALD groups.

## 3 RESULTS

**FIGURE 1:**
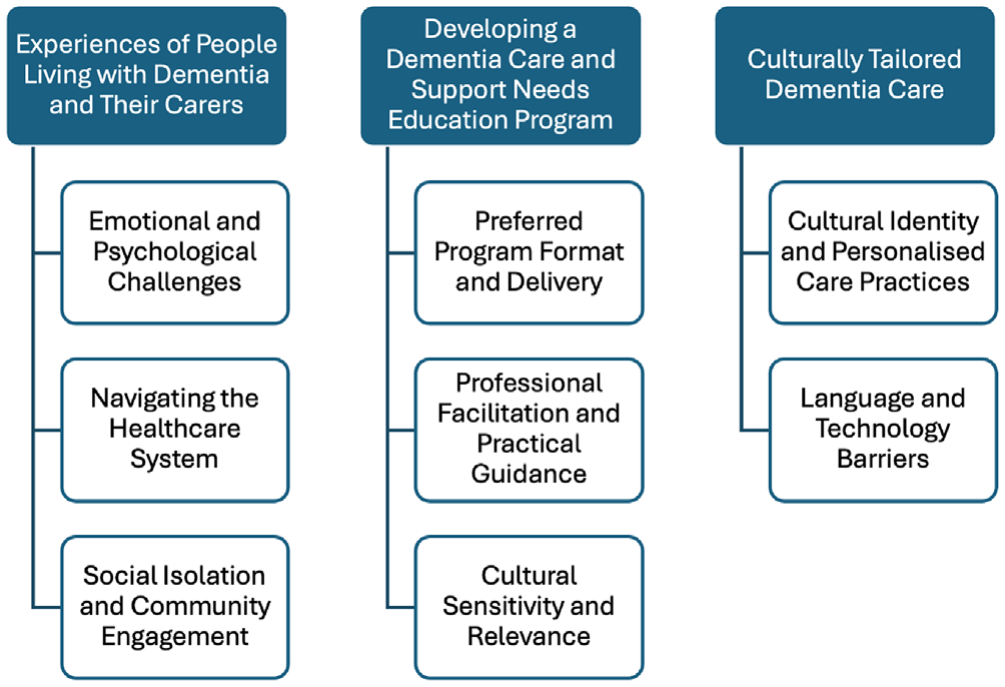
Main themes and sub‐themes from thematic analysis.

A total of 36 individuals participated in the study, including nine individuals living with dementia and 27 caregivers. The caregivers consisted of both immediate and extended family members. Table 2 provides a detailed summary of participants demographics. The study involved three focus groups with the Macedonian community, held at a local community centre, and five individual or family group interviews with Italian participants, held in their homes. Thematic analysis identified the following three main themes (Figure 1).

**TABLE 2:** Participant demographics.

Community group	Macedonian	Italian
Total participants	25	11
Females	17	9
Males	8	2
People living with dementia	8	1
Years with dementia (range)	.5–20 years	4–13 years
Carers of people living with dementia	17	10
Spousal carers	5	5
Adult daughter/son	7	2
Extended family (e.g. sibling and nephew)	5	3
Age (range)	23–83	44–93
Duration of caregiving (range)	6 months–20 years	4–12 years

### 3.1 Experiences of people living with dementia and their carers

#### 3.1.1 Emotional and psychological challenges

Participants from the Macedonian and Italian communities described a range of emotional struggles upon learning of their loved one's dementia diagnosis. Initial reactions commonly included fear, confusion and feeling unprepared. Many felt overwhelmed and uncertain about how to proceed. As one Macedonian family caregiver shared:

When I first found out about my mother, I got scared and I didn't know what to ask the doctor. (Macedonian, Family caregiver, 66)

Denial and resistance from individuals with dementia posed significant challenges for families. This stemmed from frustration and a desire to maintain a sense of independence. A Macedonian caregiver described the difficulty his father had in understanding his condition:

He (My father) would never accept that he had dementia. He would say: ‘Are you forcing me to say I'm stupid? I know I'm all right’ But he wasn't. (Macedonian, Family caregiver, 72)

Family caregivers often underestimated the progressive nature of dementia, which added to their emotional strain. One remarked:

We didn't expect that it would get worse. (Macedonian, Family caregiver, 78)

Adapting to the evolving needs of a loved one with dementia required ongoing emotional resilience and learning. As one Italian family caregiver described:

The person she is closest to (the person living with dementia) has to understand these things, things that aren't easy, but I am learning every day how to adapt to a person with dementia. (Italian, Family caregiver, 81)

#### 3.1.2 Navigating the health‐care system

Caregivers reported significant barriers in accessing appropriate health‐care and support services, often exacerbated by language difficulties and cultural differences. While some participants had positive experiences with proactive health‐care providers, others struggled to receive adequate guidance. One Italian caregiver shared a positive encounter:

The GP was very good, he had already picked it up in previous visits, even the secretary at the GP knowing them, even knowing them for 50 years going back to the same GP all the time. (Italian, Family caregiver, 54)

Conversely, others described negative experiences, particularly with specialists who failed to provide comprehensive information on disease progression and available support:

For mum more than me, the questions would have been, you know, what can we do, where is this going, what help can I get and we didn't get any of that. (Italian, Family caregiver, 74)

Many participants highlighted low health literacy as a barrier to accessing services, often due to limited English proficiency following migration. One caregiver emphasised:

If there is more information in Macedonian, it would be a lot easier for people to understand. (Macedonian, Family Caregiver, 24)

#### 3.1.3 Social isolation and community engagement

Participants expressed a strong desire for greater social engagement and community support to reduce the isolation experienced by individuals with dementia and their caregivers. One participant living with dementia highlighted the importance of social interactions:

I want to meet in the community centre more often, to talk more about that, to be with a group. I don't want to be on my own at home because my husband doesn't believe me, he makes me do things around the house all the time, but I can't; he wants to see me working, not to lie down in bed, not to sit on the lounge. (Macedonian, Participant living with dementia, 68)

Another participant explained how attending a support group in Sydney helped her cope with isolation, showing the broader need for such opportunities:

I used to look forward to the group, I learned a lot of things. (Italian, Family caregiver, 81)

### 3.2 Developing a dementia care and support needs education program

#### 3.2.1 Preferred program format and delivery

Both communities preferred face‐to‐face sessions over online formats, valuing personal interaction for better understanding and emotional support. One caregiver emphasised:

The program to be face to face, not online, to be more frequently for people with dementia, twice a month, two hours, for example [Annual Illawarra] Dementia Forum. (Macedonian, Family caregiver, 66)

Printed materials, such as flyers and fact sheets, were also favoured by participants over digital resources because they provided easily accessible guidance:

Tips will help on paper, flyers, factsheets. (Macedonian, Family caregiver, 71)

Some participants, particularly older caregivers, expressed difficulties using online platforms because of language barriers and limited IT literacy. An Italian caregiver highlighted a preference for verbal, in‐person learning:

Persons who explain in person how you should behave, and I listen, and take that information on, but if you tell me to go online, it's something complicated. (Italian, Family caregiver, 81)

#### 3.2.2 Professional facilitation and practical guidance

Participants emphasised the importance of having health‐care professionals lead the education program, providing evidence‐based guidance on dementia care prevention, management and support resources. A Macedonian caregiver stressed the need for expert‐led sessions:

I would suggest to have an education program with health professionals to explain to people how to prevent it, what to do, with activities… advice for the family. (Macedonian, Family caregiver, 80)

Beyond medical insights, caregivers also wanted practical strategies for day‐to‐day dementia care. They valued structured group meetings where professionals could offer hands‐on demonstrations and advice:

Going to a group where people can meet, health professionals to come to give presentations and talks, to explain to us about dementia, things we should do, how to behave. (Macedonian, Family caregiver, 80)

Several participants stressed the need for broader community awareness to reduce stigma and improve dementia literacy:

The education should increase the awareness for everyone, not just those who are affected.(Italian, Family caregiver, 81)

#### 3.2.3 Cultural sensitivity and relevance

Ensuring cultural relevance was a key priority for participants, who highlighted the importance of integrating familiar traditions and activities into a program. Macedonian community members suggested incorporating culturally meaningful elements:

It has to be appropriate for our tradition and culture, something that is connected with our past…making simple things, cooking savoury and sweets. Music, dancing, playing cards, crosswords. (Macedonian, Participant living with dementia, 68)

Language accessibility was also a major concern. Many participants stressed the need for sessions to be conducted in their native language to ensure full comprehension and enable meaningful participation:

If this service were to be provided in the Italian language, I'd be able to follow it, but if it's done in English, it's not easy to respond, it's not easy to respond. (Italian, Family caregiver, 81)

### 3.3 Culturally tailored dementia care

#### 3.3.1 Cultural identity and personalised care practices

Maintaining cultural identity was seen as essential to dementia care, as familiar traditions and language help individuals retain a sense of self. Participants expressed that even as dementia progresses, aspects of personality, such as humour and joy can remain intact:

One positive thing is, for how bad things are, that part of his personality where he wants to make a little joke, or, he wants to say something silly, he likes to joke, that's still there. (Italian, Family caregiver, 54)

Caregivers stressed the importance of patience, empathy and love when supporting individuals with dementia, even in challenging moments:

A lot of patience and love too. You have to give love even if you are cranky. (Italian, Family caregiver, 80)

Participants advocated for dementia care programs that are culturally sensitive and tailored to the specific needs of CALD populations. One participant shared:

The program has to be in Macedonian…to be adjusted to working with Macedonian people in terms of language, culture, traditions, activities they used to do when they were young, which are completely different for someone who is from an English‐speaking background. (Macedonian, Participant living with dementia, 68)

Group‐based activities that encourage social connection were highly valued, with participants suggesting excursions, cultural events and communal pastimes, such as gardening and music:

It would be good to go to a group to socialise with other people, older people need gatherings, music, communication with each other, to go out, to go for a walk, to take trips to the zoo… to do gardening… (Macedonian, Participant living with dementia, 74)

#### 3.3.2 Language and technology barriers

English language proficiency and IT literacy were significant barriers to accessing dementia care resources. Many older CALD individuals struggled to understand resources written in English as well as digital resources:

But for a person like me, and others my age we don't know much English, don't know how to read, don't know how to use all this, that's not going to help. (Italian, Family caregiver, 81)

I can't use a computer. I can't read and write in English and I can't speak properly. (Macedonian, Participant living with dementia, 74)

Participants appreciated when health‐care professionals communicated in their native language, highlighting the importance of linguistic accessibility in health‐care settings:

I'm very thankful to the Psychiatrist who explained to me in our language. (Macedonian, Family caregiver, 71)

## 4 DISCUSSION

This study provided valuable insights into the dementia care and support needs of the Macedonian and Italian communities in the Illawarra Shoalhaven region. Both communities emphasised the significant emotional impact of a dementia diagnosis. While feelings of fear, denial and uncertainty were common, cultural beliefs further discouraged open discussions about dementia. Stigma remained a significant barrier in many CALD communities, often delaying diagnosis and limiting engagement with support services.^17^ In Mediterranean and Eastern European cultures, caregiving is traditionally viewed as a family responsibility, with a strong preference for informal care over formal services.^18^ However, as caregiving demands grow, these expectations can become unsustainable. Addressing dementia care in CALD communities requires not only supporting caregivers but also actively reducing stigma and misinformation.^19^

Language barriers and low health literacy remain significant obstacles for CALD communities in accessing health‐care and support services. Miscommunication with health‐care providers can lead to delays or inappropriate care, contributing to health disparities.^20^ While materials translated into different languages and culturally sensitive service delivery are recommended, these measures alone are insufficient.^21^ Relational trust is essential for CALD individuals, who face additional barriers, such as discrimination, past negative experiences and a lack of culturally competent care.^22^ In this study, participants who had established trust with their health‐care providers felt more comfortable seeking help, whereas those without such connections were hesitant to engage with the system. The need for participants to seek support outside Illawarra highlighted the ongoing gaps in local dementia care services and reinforced the importance of this research. Broader studies further highlighted that relational, community‐based engagement, where health‐care providers built and maintained ongoing connections with CALD communities, fostered trust. This approach reduced the burden of repeatedly explaining medical histories and ultimately improved health‐care engagement, satisfaction and outcomes.^23^, ^24^

Social isolation emerged as another significant issue, particularly for caregivers and those living in regional areas. While many people with dementia preferred face‐to‐face interactions, research showed this preference is even stronger in CALD communities due to language barriers, cultural differences and limited digital literacy.^25^ As a result, digital solutions might be less effective for some CALD groups. Participants in this study emphasised the need for community‐based programs that provide both practical dementia care education and opportunities for social engagement. Previous research demonstrated the effectiveness of such programs, showing that tailored, in‐person initiatives enhanced dementia awareness, strengthened social connections and fostered a sense of belonging.^26^ Other research highlighted the role of culturally responsive, face‐to‐face support in reducing isolation and improving the accessibility of dementia education.^27^ These findings reinforced the need for approaches that integrate both social and educational components to better support CALD communities.

Both communities strongly emphasised the need for regular, culturally tailored dementia education programs. Participants recommended that these programs be led by culturally competent health‐care professionals with expertise in both dementia care and the specific cultural backgrounds of the communities. Our findings support a previous study that found culturally responsive education enhanced engagement and service uptake among CALD populations.^28^ However, this recommendation might not be possible because of the availability and training of such professionals. There are workforce challenges in delivering culturally competent dementia care.^29^ One potential solution is to establish partnerships with bilingual community health‐care workers, who can help bridge the gap between mainstream services and CALD families.^30^

### 4.1 Strengths and limitations

A key strength of this study was its co‐design approach, which incorporated the lived experiences of the target communities to develop practical recommendations for a tailored dementia care framework. Community members actively validated and refined results, ensuring cultural relevance and accuracy. Selection bias might have occurred due to the reliance on specific community networks known to the research team for recruitment of participants. This potentially excluded individuals from local Italian and Macedonian groups with differing views who were not engaged with the participating community groups. The uniqueness of these groups limits the transferability of results to other cultural groups.

## 5 CONCLUSIONS

This study highlighted the complex challenges Macedonian and Italian communities face in accessing dementia care, emphasising the need for culturally tailored, community‐led solutions. The next key step is piloting a dementia education program within these communities to assess its impact on awareness and service access. Findings from this pilot can inform adaptations for broader CALD communities across the state. Importantly, it cannot be underestimated that advancing equitable dementia care requires sustained investment in culturally competent education, workforce training and strong community partnerships.

## FUNDING INFORMATION

This study was supported by funding contributions from the University of Wollongong.

## CONFLICT OF INTEREST STATEMENT

No conflicts of interest declared.

## Data Availability

The data that support the findings of this study are available on request from the corresponding author. The data are not publicly available due to privacy or ethical restrictions.
